# RNA-Seq and CyTOF immuno-profiling of regenerating lacrimal glands identifies a novel subset of cells expressing muscle-related proteins

**DOI:** 10.1371/journal.pone.0179385

**Published:** 2017-06-29

**Authors:** Dillon Hawley, Jian Ding, Suharika Thotakura, Scott Haskett, Hema Aluri, Claire Kublin, Audrey Michel, Lisa Clapisson, Michael Mingueneau, Driss Zoukhri

**Affiliations:** 1 Depratement of Comprehensive Care, Tufts University School of Dental Medicine, Boston, Massachusetts, United States of America; 2 Immunology Research, Biogen, Cambridge, Massachusetts, United States of America; 3 Department of Ophthalmology, Tufts University School of Medicine, Boston, Massachusetts, United States of America; University of Minnesota Medical Center, UNITED STATES

## Abstract

The purpose of the present studies was to use CyTOF and RNA-Seq technologies to identify cells and genes involved in lacrimal gland repair that could be targeted to treat diseases of lacrimal gland dysfunction. Lacrimal glands of female BALB/c mice were experimentally injured by intra-glandular injection of interleukin 1 alpha (IL-1α). The lacrimal glands were harvested at various time points following injury (1 to 14 days) and used to either prepare single cell suspensions for CyTOF immuno-phenotyping analyses or to extract RNA for gene expression studies using RNA-Seq. CyTOF immuno-phenotyping identified monocytes and neutrophils as the major infiltrating populations 1 and 2 days post injury. Clustering of significantly differentially expressed genes identified 13 distinct molecular signatures: 3 associated with immune/inflammatory processes included genes up-regulated at days 1–2 and 3 associated with reparative processes with genes up-regulated primarily between days 4 and 5. Finally, clustering identified 65 genes which were specifically up-regulated 2 days post injury which was enriched for muscle specific genes. The expression of select muscle-related proteins was confirmed by immunohistochemistry which identified a subset of cells expressing these proteins. Double staining experiments showed that these cells are distinct from the myoepithelial cells. We conclude that experimentally induced injury to the lacrimal gland leads to massive infiltration by neutrophils and monocytes which resolved after 3 days. RNAseq and immunohistochemistry identified a group of cells, other than myoepithelial cells, that express muscle-related proteins that could play an important role in lacrimal gland repair.

## Introduction

The inability of the lacrimal gland to repair/regenerate as a result of chronic inflammation, leads to lacrimal gland dysfunction. This chronic inflammation is associated with several pathological conditions including several autoimmune diseases such as Sjögren’s syndrome, sarcoidosis, and rheumatoid arthritis [1, 2]. The lacrimal gland produces the majority of the aqueous portion of the tear film. Thus, dysfunction of the lacrimal gland results in decreased secretion of the aqueous layer of the tear film leading to keratoconjunctivitis sicca (KCS) or dry eye disease [3, 4]. Dry eye symptoms include irritation, blurred and fluctuating vision and if unmanaged can lead to sight-threatening corneal ulceration in severe cases. The overall economic burden of dry eye disease in the US is estimated at $5 billion annually due to reduced productivity and the cost of therapies. Only two FDA approved drugs, with limited efficacy, are currently available stressing the need for additional therapies for dry eye disease.

Previously, we have shown that the murine lacrimal gland has the ability to repair itself after experimentally induced injury [5]. Inflammation caused by the injury leads to transient aqueous tear deficiency, which is relieved, after repair is complete in 7–14 days. The exact mechanisms that underlie lacrimal gland repair are still not fully understood. It has been shown that lacrimal gland injury/inflammation triggers epithelial-mesenchymal transition (EMT), which generates cells with mesenchymal stem cell-like properties [6]. The mobilization of slowly-cycling, label-retaining cells as well as resident stem/progenitor cells during lacrimal gland repair has also been demonstrated [7]. In addition, runt-related transcription factor 1 and 3 (Runx1 and 3), regulators of stem cell proliferation and differentiation, are also involved in lacrimal gland repair [8].

Novel technologies such as RNA-sequencing (RNA-Seq) and cytometry by time-of-flight (CyTOF or mass cytometry) allow for the interrogation of the whole transcriptome in an unbiased way and deep immunophenotyping of heterogeneous single cell suspensions, respectively. Mass cytometry has been used to characterize immune subsets in the target tissue and blood of both mouse models of Sjögren’s syndrome and patients with primary Sjögren’s syndrome [9, 10]. RNA-Seq transcriptomic analyses were reported in various diseases, such as Crohn’s disease, renal injury, chronic periodontitis, and many others [11–14]. Differential gene expression analysis of RNA-Seq and microarray data has become a common tool to compare gene expression between individuals, tissues, and cell subsets. A few studies used microarrays to investigate gene expression in the lacrimal gland following acute corneal trauma, loss of parasympathetic secretion and botulinum toxin B injury [15–17]. RNA-seq has not been used yet to investigate gene expression changes occurring during lacrimal gland tissue repair following an inflammatory injury process.

Herein, we used CyTOF immunophenotyping and RNA sequencing technologies to investigate the processes and cells involved in lacrimal gland repair in a model of experimentally-induced lacrimal gland tissue injury. Among 40,000 coding DNA sequences examined, 2,131 transcripts showed statistically significant expression changes upon tissue injury when compared to un-injured lacrimal glands. Both CyTOF and RNA-Seq analyses identified neutrophils and monocytes as the main infiltrating immune cell types 1 and 2 days post injury. Following resolution of the inflammatory process, the expression of a small number of genes was significantly altered between days 3 and 5 despite the major histological alterations that occurred during this time period. Finally, we identified a group of cells expressing muscle-related proteins 2 days post injury that might play a role in lacrimal gland regeneration. Interestingly, these cells did not express typical markers associated with myoepithelial cells, the only known cells in the lacrimal glands to express muscle proteins, thereby indicating that they might correspond to a novel subset of cells or a novel cellular phenotype.

## Materials and methods

### Animals and treatments

BALB/c mice (10–12 weeks old) were purchased from The Jackson Laboratories (Bar Harbor, ME). The lacrimal glands of each mouse (3 mice / treatment group) were injected with either saline (control) or 1 μg interleukin-1 alpha (IL-1, PeproTech, Rocky Hill, NJ) in a total volume of 2 μL. The lacrimal glands were harvested 1, 2, 3, 4, 5, 7, or 14 days post-injection. The lacrimal glands from non-injected mice were also harvested and used as normal control. All experiments were performed in compliance with the ARVO Statement for the Use of Animals in Ophthalmic and Vision Research and the Guidelines for the Care and Use of Laboratory Animals published by the US National Institutes of Health (NIH Publication No. 85–23, revised 1996) with approval from the Tufts Medical Center Animal Care and Use Committee.

### RNA extraction, quality control, and RT-PCR

RNA was extracted from the lacrimal glands using the miRNeasy isolation kit (Qiagen, Valencia, CA), using the manufacturer’s protocol. Briefly, the tissue was homogenized in QIAzol lysis buffer and incubated for 5 minutes at room temperature. Chloroform was then added to the homogenate, followed by centrifugation at 12,000 x g at 4°C. Next, the aqueous layer was mixed with 1.5 volumes of absolute ethanol and run through the MiniElute spin columns. Buffers RWT, RPE (both provided in kit), and 80% ethanol were then sequentially run through to wash the column. Finally, 20 μL of RNAse-free distilled water was used to elute RNA from the column. RNA samples were stored at -80°C until downstream use.

The concentration and purity of RNA was assessed using a NanoDrop 1000 (ThermoFisher Scientific, Waltham, MA). Total RNA quality was assessed by examination of RNA size distribution on RNA Nano LabChips (Agilent Technologies, Santa Clara, CA) processed on the Agilent 2100 Bioanalyzer (Agilent Technologies, Santa Clara, CA), using the total RNA electrophoresis program. For samples analyzed, a RNA integrity number (RIN) was generated for each Bioanalyzer trace using 2100 Expert Software (Agilent Technologies, Santa Clara, CA). The maximum RIN score is 10.0 and samples with RIN values of 8.0 or higher were viable for RNA-sequencing analyses.

Purified total RNA (20 ng) was used for reverse transcription and PCR amplification with OneStep RT-PCR Kit (Qiagen, Valencia, CA) using primers designed using NCBI/ Primer-BLAST specific to *Vimentin*, *Snai1*, *Runx2*, and *GAPDH* in a thermal cycler (2720 Thermal Cycler; Applied Biosystems, Foster City, CA). The reverse transcription reaction was conducted at 52°C for 30 minutes followed by PCR according to the manufacturer's instructions. The cycling conditions were 15 minutes hot start at 95°C, 25 to 30 cycles of denaturation for 40 seconds at 94°C, annealing for 40 seconds at 53°C, extension for 1 minute at 72°C, and a final extension at 72°C for 10 minutes. After amplification, the products were separated by electrophoresis on a 1.5% agarose gel and visualized by UV light after ethidium bromide staining.

### RNA-Seq library preparation and sequencing

For each lacrimal gland sample, 500 ng of RNA were used to construct sequencing libraries using Illumina’s TruSeq Stranded mRNA HT Sample prep kit (Cat # RS-122-2103). The products were purified and enriched with PCR for 12 cycles to create the final cDNA library. Automated library preparations were carried out according to manufacturer’s recommendations using an Arrayplex automated liquid handler (Biomek FXP Arrayplex, Beckman Coulter). TruSeq adapter sequences available in the Illumina Customer Sequence Letter on Illumina support were used in the experiment. Libraries were quantified using Illumina Library Quantification Kit (KK4824). Multiplexed samples were equimolarly pooled, diluted to 2nM pool for final analysis on Agilent High Sensitivity DNA Kit to run on three rapid flowcells per lane and sequenced using 2 x 50 ntd paired-end runs of Illumina HiSeq 2500 platform.

### RNA-Seq mapping, quality control, and differential expression analysis

STAR aligner was used for genome alignment and to generate BAM files. The percentage of uniquely mapped reads was used as a metric to identify poor sequencing quality and exclusion of samples from gene quantification and differential expression analysis. RSEM software was then used for gene expression quantification and principal component and heatmap analyses were performed to identify biologically reproducible replicates for downstream differential expression analysis using DESeq2.

### Statistical analyses

All statistical analyses and plotting were performed using the R statistical programming language (version 3.2.2; www.r-project.org). Significantly up- and down-regulated genes were identified based on a fold change (FC) of +2 or -2, respectively, and a false discovery rate corrected p-value of 0.05 or less. Raw expression files were used to identify genes that were not expressed in uninjured lacrimal glands based on an expression value of zero in all three replicate samples. The expression patterns of differentially expressed genes, fold-fold change plots, and cluster plots were created, using the ggplot2 package (version 2.2.1; https://cran.r-project.org/web/packages/ggplot2/index.html). The KMeansClustering module of GenePattern (version 3.9.8; The Broad Institute, Cambridge, MA) was used to perform clustering and identify molecular signatures of differentially expressed genes using default settings and k = 50 clusters (where k ≈ √# genes). Differentially expressed genes lists from each day and clusters were used for gene set enrichment analysis of biological process gene ontology terms using the gProfileR package (version 0.6.1; https://cran.r-project.org/web/packages/gProfileR/index.html). The following settings were used for enrichment analyses to identify significant hits: organism = “mmusculus”, src_filter = “GO:BP”, hier_filtering = “moderate”, and max_p_value = 0.001.

### Preparation of lacrimal gland single cell suspensions

Excised lacrimal glands were minced using scissors to dissociate the tissue and enzymatically digested at 37°C under rotating agitation (100 rpm) in DMEM medium containing 232 U/mL collagenase II (Worthington, Lakewood, NJ) and 8 U/mL DNAse I (Sigma-Aldrich, Natick, MA). After two washes in calcium- and magnesium-free phosphate buffered saline (PBS) containing 1mM EDTA, cellular aggregates were resuspended in 0.5 mL of TrypLE Express Enzyme (Gibco, ThermoFisher Scientific, Waltham, MA) and incubated for 2 minutes at 37°C. Enzyme inactivation was achieved by dilution with 4 mL of DMEM and immediately followed by gentle up-and-down pipetting to facilitate cell dissociation. The resulting cell suspension was washed twice with medium supplemented with 0.8 U/mL DNAse I. Single cell suspensions for CyTOF immuno-phenotyping experiments were resuspended at 10x10^6^ cells/mL in PBS and fixed in 2% methanol-free formaldehyde (Electron Microscopy Sciences, Hatfield, PA) for 20 minutes at room temperature. After 2 washes in PBS-0.5% BSA-0.02% sodium azide, 100 μL cell aliquots containing a maximum of 2 x 10^6^ cells each were flash frozen on dry ice and stored at -80°C prior to use. Spleens and lymph nodes were disaggregated into single cell suspensions and red blood cells were lysed using RBC lysis buffer (eBiosciences, San Diego, CA).

### Mass cytometry

Fixed and frozen cell suspensions were thawed on ice. Samples were stained and prepared for CyTOF analysis as previously-described [18], using an optimized cocktail of 30 metal-conjugated antibodies designed to identify major and minor mouse cell subsets (Table 1). Following acquisition on a CyTOF II (Fluidigm, San Francisco, CA), samples were normalized to internal bead standards. viSNE algorithm was used to define an optimal gating hierarchy and to explore tissue cellular composition in an unbiased manner. Cell subsets were next identified by manual gating using FlowJo software.

**Table 1:** Mass cytometry (CyTOF immunophenotyping) antibody panel.

Antigen	Antibody Clone	Symbol	Mass
CD8	53–6.7	Cd	114
Ly-6G	RB6-8C5	Pr	141
CD11c	N418	Nd	142
CD115	AFS98	Nd	144
CD4	RM4-5	Nd	145
F4/80	BM8	Nd	146
CD45.2	104	Sm	147
CD19	6D5	Sm	149
IgD	11-26c.2a	Nd	150
IgM	RMM-1	Eu	151
CD3ε	145-2C11	Sm	152
CD45.1	A20	Eu	153
CD11b	M1/70	Sm	154
Thy1.2	30-H12	Gd	156
CD93	AA4.1	Gd	158
CD23	B3B4	Tb	159
CD5	53–7.3	Gd	160
Ly-6C	HK1.4	Dy	162
CD25	PC61	Dy	164
CD317	927	Ho	165
CD326	G8.8	Er	166
NKp46	29A1.4	Er	167
CD21	7G6	Er	168
TCRβ	H57-597	Tm	169
CD49b	HMα2	Er	170
CD44	IM7	Yb	171
CD86	GL1	Yb	172
IA-IE	M5/144	Yb	174
CD38	90	Lu	175
B220	RA3-6B2	Yb	176
Cisplatin		Pt	195

### Immunohistochemistry

Excised lacrimal gland tissue was fixed in 4% formaldehyde at 4°C overnight followed by paraffin embedding. Tissue sections (6 μm) were mounted on microscope slides for histopathology and immunofluorescent staining. Sections were de-paraffinized and re-hydrated through graded alcohol solutions. For histopathology experiments, tissue sections were stained with Harris’ Hematoxylin and Eosin Solutions. For immunofluorescence experiments, tissue sections were subjected to heat-mediated antigen retrieval, for 15 minutes, using Antigen Unmasking Solution (VectorLabs, Burlingame, CA). Next, sections were permeabilized using 0.1% Triton X-100 for 10 minutes at room temperature. Nonspecific binding sites were blocked using 10% normal donkey serum and donkey anti-mouse IgG blocking antibodies, for mouse primary antibodies (Jackson ImmunoResearch Laboratories, Westgrove, PA). The slides were then incubated overnight at 4°C with one of the following primary antibodies: from Abcam (Cambridge, MA), a mouse monoclonal antibody against ryanodine receptor 1 (1:100) and rabbit polyclonal antibodies against alpha smooth muscle actin (1:250) and collagen I (1:300); from eBiosciences (San Diego, CA) a mouse monoclonal antibody against desmin (1:100); from Santa Cruz Biotechnologies (Santa Cruz, CA) a mouse monoclonal antibody against β-taxilin (1:100) and sarcalumenin (1:100). Sections without primary antibody were included as negative controls. Next, sections were incubated with TRITC or FITC conjugated secondary antibodies against the appropriate species (1:100; Jackson ImmunoResearch Laboratories, Westgrove, PA) for 60 minutes at room temperature. For double labeling experiments staining was completed sequentially with the first set of primary and secondary antibody staining before starting incubation with the second antibody. Finally, sections were covered with VectaShield Antifade Mounting Medium containing DAPI (VectorLabs, Burlingame, CA) to counterstain nuclei. Sections were visualized using a microscope equipped for epi-illumination (Nikon UFXII Epi-Illuminator).

## Results and discussion

### Acute lacrimal gland injury

**Fig 1:**
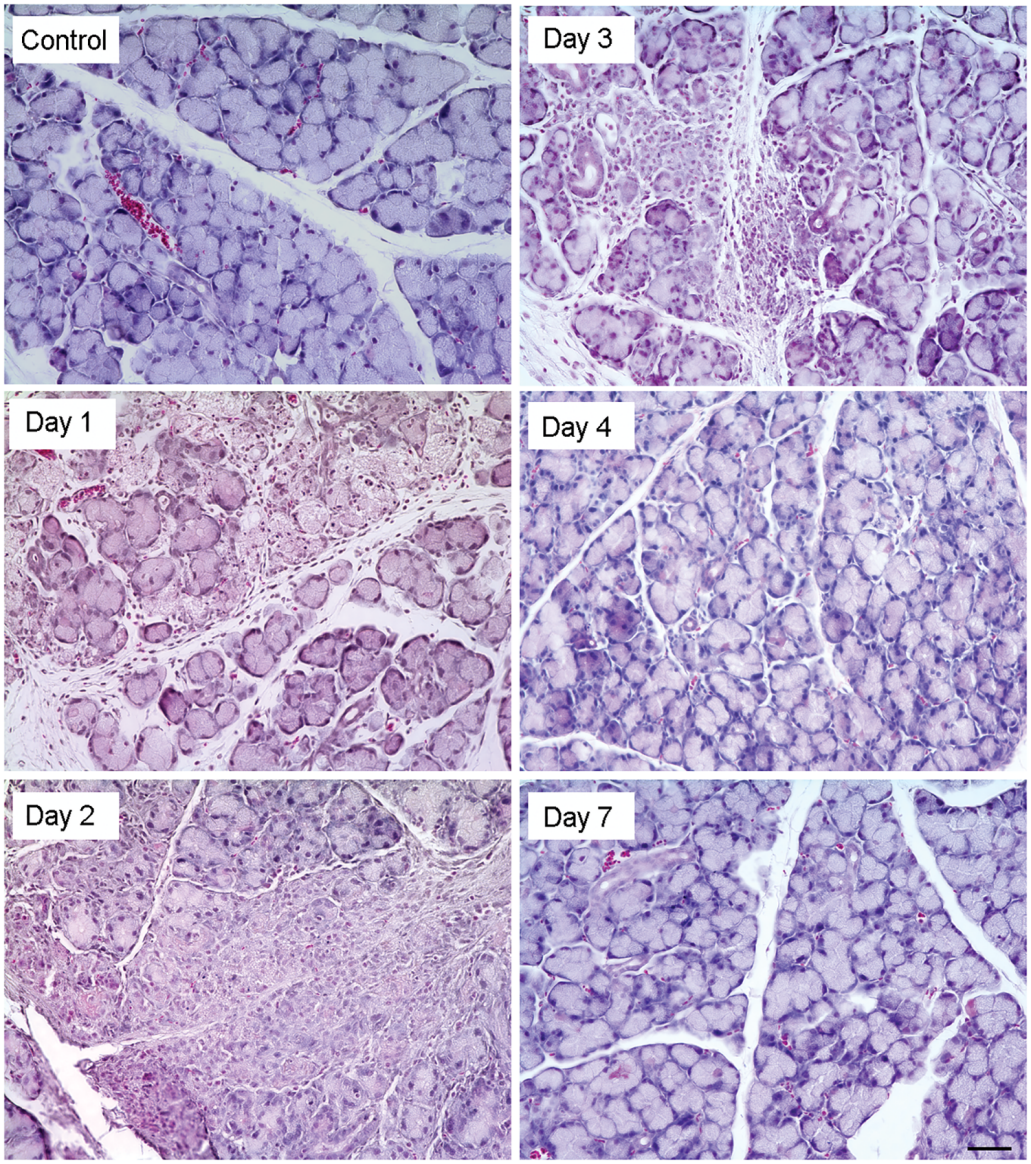
Histological analysis of IL-1α injected lacrimal glands. Lacrimal gland tissue was excised 1, 2, 3, 4, and 7 days post IL-1α or saline (vehicle) injection and processed for hematoxylin and eosin staining. Scale bar represents 50 μm for all panels.

We have used our established animal model of acute injury to the lacrimal gland via intraglandular injection of IL-1α. In this model [19], the lacrimal gland undergoes major histological changes in response to IL-1α induced injury as evidenced by the large amount of infiltrating immune cells 1, 2, and 3 days post injury and extensive tissue damage (Fig 1). Repair begins 2–3 days post injury resulting in histologically normal tissue starting at day 4 (Fig 1). In our previous work, we reported that lacrimal gland injury involves activation of the epithelial to mesenchymal transition (EMT) as evidenced by up-regulation of *Snai1* and *Vimentin* gene expression [6]. Following total RNA extraction and quality control, as described in the Methods section (see RNA integrity values in S1 Table), the expression of *Snai1* and *Vimentin* was assessed to identify the gene signature associated with lacrimal gland injury and repair. As previously reported [6], we found that *Snai1* and *Vimentin* gene expression was up-regulated 1 and 2 days post injury and returned to basal levels after 5–7 days when tissue repair is completed (data not shown).

**Fig 2:**
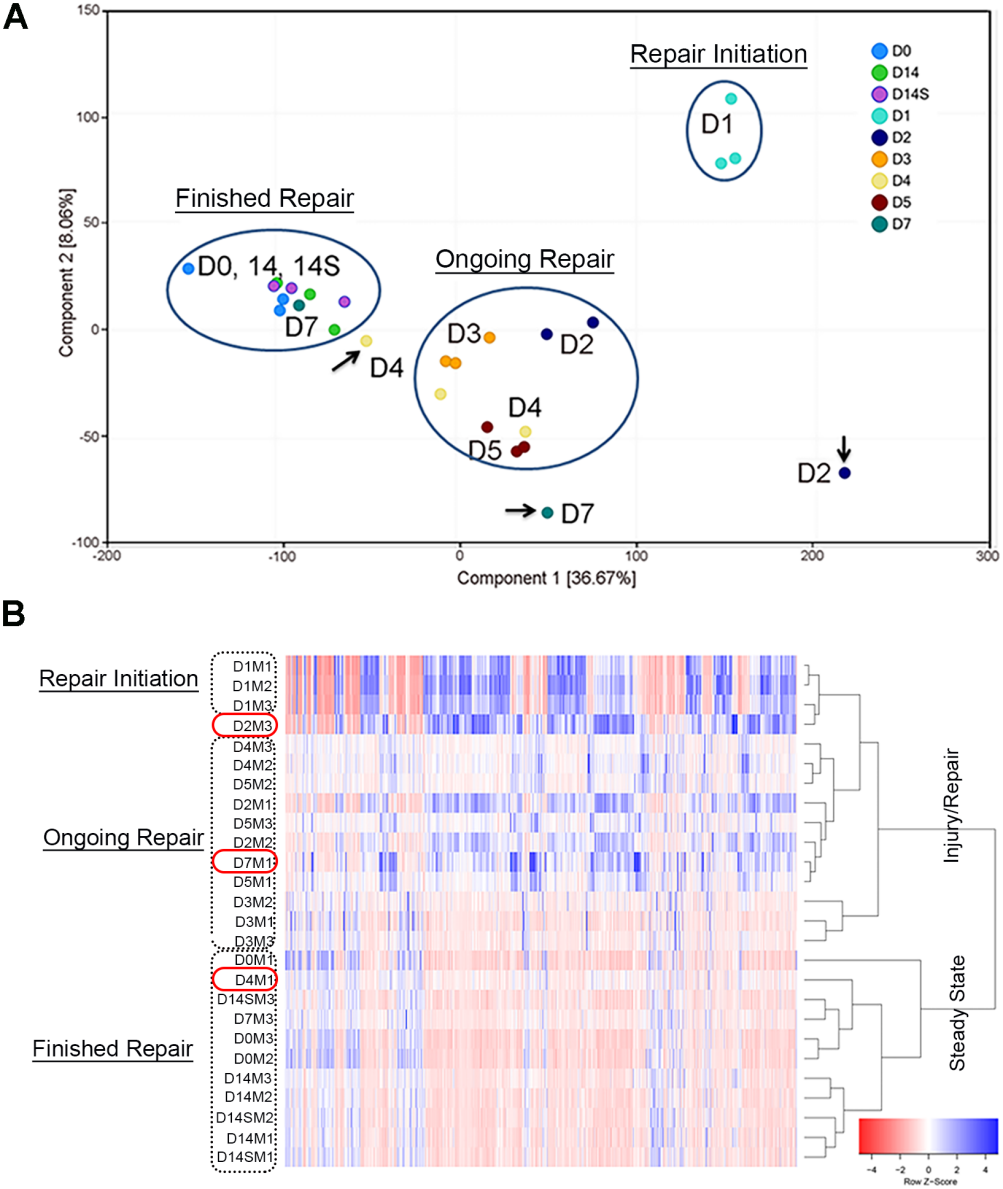
Principal component and heatmap analyses of RNA-sequencing samples. All samples were quality controlled to identify samples for exclusion from differential expression analysis. (A)Principal components analysis (PCA) was used to investigate replicate variability with 3 distinct clusters successfully identified. (B) Heatmap analysis was also used to cluster samples to identify samples for exclusion which identified 4 distinct clusters of samples. Samples (arrows in A, red circles in B) outside the defined clusters were excluded from downstream analyses.

Transcriptomic analysis of the lacrimal glands at different time points following IL-1α injury was performed. The percentage of uniquely mapped paired reads was >80% in all samples, except one sample which was therefore excluded. Principal component analysis of the remaining samples showed that the first two principal components explained approximately 45% of the variability in the dataset (Fig 2A). Samples formed three distinct clusters in the PCA plot. Day 1 samples clustered together and were the farthest away from the other samples indicating extensive changes in gene expression during initiation of the reparative phase. Most of the samples from injured lacrimal glands at day 2, 3, 4, and 5 clustered together and corresponded to the ongoing tissue repair process, which, based on our previous work, occurs during this time period. Finally, samples from injured lacrimal glands at day 14 clustered with control samples (uninjured lacrimal glands and saline-injected lacrimal glands at day 14 (sample 14S), indicating return to steady-state gene expression characteristic of healthy lacrimal gland tissue. Heatmap analysis also revealed a similar pattern with 2 large clusters distinguishing steady-state samples from injury/repair samples (Fig 2B). Within the injury/repair cluster we also identified 3 separate clusters, day 1 samples form an initiation of repair cluster, day 2, 4, and 5 samples form an ongoing repair process cluster, and day 3 samples form a distinct cluster. Although the day 3 samples do not cluster with the other samples reminiscent of ongoing repair they do fall within the injury/repair cluster and not the steady-state cluster. Three outlier samples in the PCA analysis (arrows in Fig 2A) and heatmap (red boxes in Fig 2B) were identified and excluded from differential expression analyses. These samples did not cluster within the expected groups and may reflect biological variability that occurs in vivo following tissue injury.

The above data show that the major histological changes that are observed in response to experimentally induced injury with IL-1α are reflected in the RNA-sequencing data. The distinct clusters identified by PCA and heatmap analysis, correlate with the observed timeline of tissue inflammation and repair.

### Global changes in gene expression

**Fig 3:**
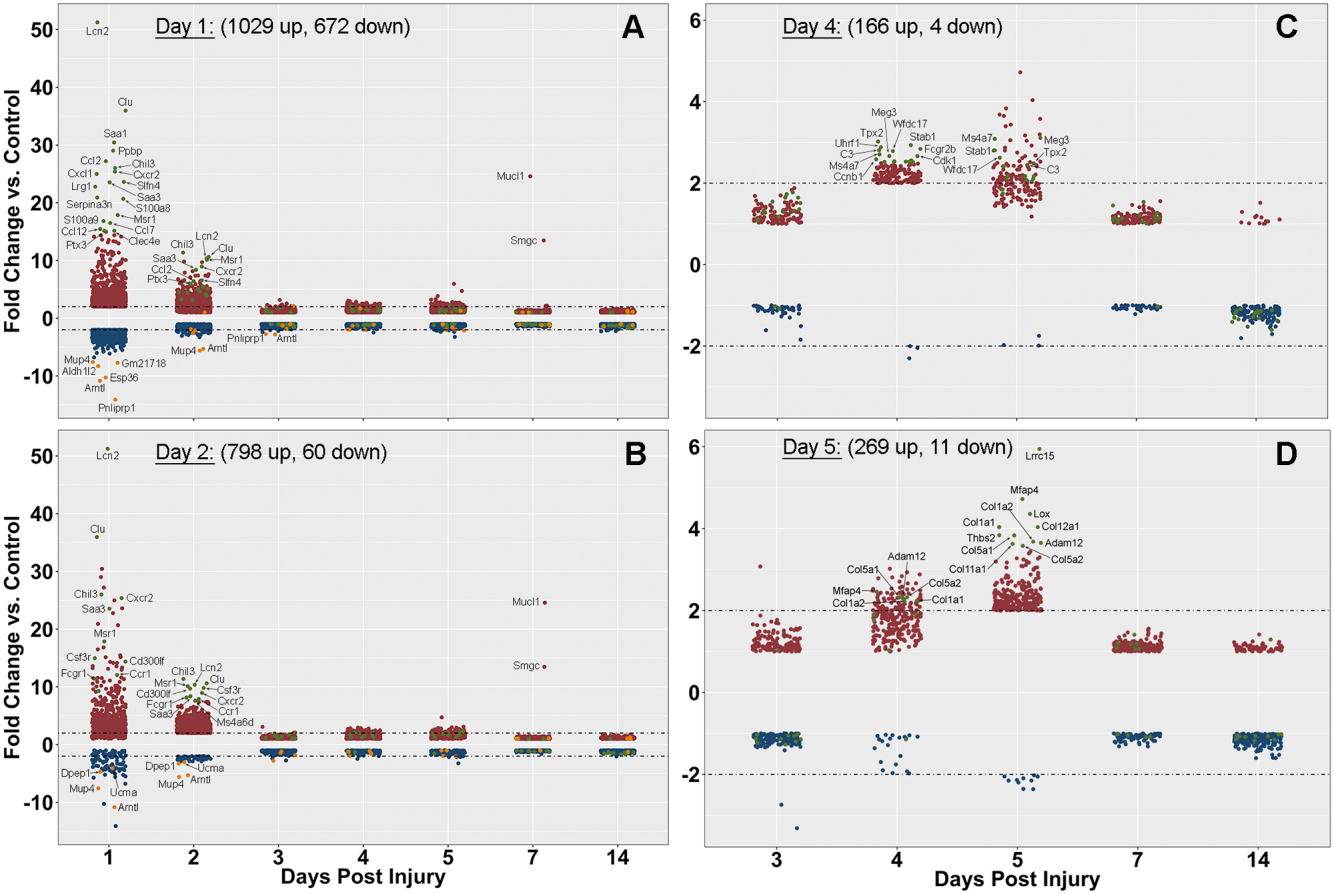
Expression pattern of significantly differentially expressed genes by day. The significantly differentially expressed genes at days 1, 2, 4, and 5 were plotted to visualize the global expression of these genes across all time points (days 3, 7, 14 and 14S data not plotted based on the small number of genes). Day 4 and 5 significantly differentially expressed genes exclude day 1 and 2 expression for visualization purposes (very few genes up/down-regulated). Data points colored based on regulation at the day of interest; green = strongly up-regulated, red, up-regulated, orange = strongly down-regulated, blue = down-regulated.

In injured lacrimal glands, among 43,346 Coding DNA sequences in the mouse transcriptome, 2,131 were significantly differentially expressed when compared to control uninjured glands in at least one of the time points analyzed. Expression of 1,029, 798, 31, 166, 269, 7 and 0 genes were up-regulated at day 1, 2, 3, 4, 5, 7 and 14, respectively, while the expression of 672, 60, 13, 4, 11, 0, and 5 genes were down-regulated at day 1, 2, 3, 4, 5, 7 and 14, respectively. The expression of differentially expressed genes (DEGs) at day 1, 2, 4, and 5 was plotted at all time points to visualize their behavior during the injury and repair process. Fig 3A and 3B showed a strong up and down-regulation of numerous genes at day 1 and 2, whereas, a smaller number of genes were differentially expressed at day 4 and 5 (Fig 3C and 3D). Gene set enrichment analyses, used to identify relevant biological processes, identified genes associated with immune processes and inflammation at day 1 and 2 (S2 Table). Top enriched categories included “defense response” and “immune system process”, with p-values of 3.66x10^-69^ and 7.05x10^-91^, respectively. For example, *Lipocalin* (*Lcn2*), which is expressed by neutrophils, was the most up-regulated gene (by 52-fold on day 1 and 10-fold on day 2) (Fig 3A and 3B). On day 4 gene set enrichment identified cell cycle and extracellular matrix associated processes with a top enriched category of “mitotic cell cycle process” (p = 2.33x10^-19^) (S1 Table). Accordingly, many of the most up-regulated genes at day 4 were indeed associated with cell cycle functions such as *Tpx2* (microtubule nucleation factor) and *Uhrf1* (ubiquitin-like containing PHD and RING finger domains) which are both regulators of the mitotic process (Fig 3C). At day 5, extracellular matrix associated genes were enriched with a top enriched category of “extracellular matrix organization” (p = 2.11x10^-26^) (S2 Table). Nearly all of the strongly up-regulated genes were associated with extracellular matrix organization/deposition: numerous collagen chains (*Col1a1*, *Col1a2*, *Col12a1*, *etc*.), matrix metalloproteinases (MMPs), and other matrix remodeling genes (Fig 3D).

In this experimental model day 3 is thought to be the critical time point during which there is a switch between inflammatory and regenerative processes; there were, however, only a small number of DEGs at this time point. Of the 31 genes that were up-regulated at day 3 one third of them had no known function. The remaining genes did not have obvious function in tissue regeneration or resolution of inflammation with the exception of, *Scd2* and *Sqle*, which are associated with lipid metabolism and could be involved in the inflammatory process or its resolution. Expression of the *Sqle* gene was reported to be upregulated in monocytes stimulated with colony-stimulating factor-1 [20]. In addition, the gene *Zbtb16*, a transcription factor associated with progression of the cell cycle [21], was down-regulated by 3-fold at day 2 before being up-regulated by 3-fold at day 3 and 5. Similarly, of the 13 genes that are down-regulated none of them seem to be involved in tissue regeneration or resolution of inflammation.

**Fig 4:**
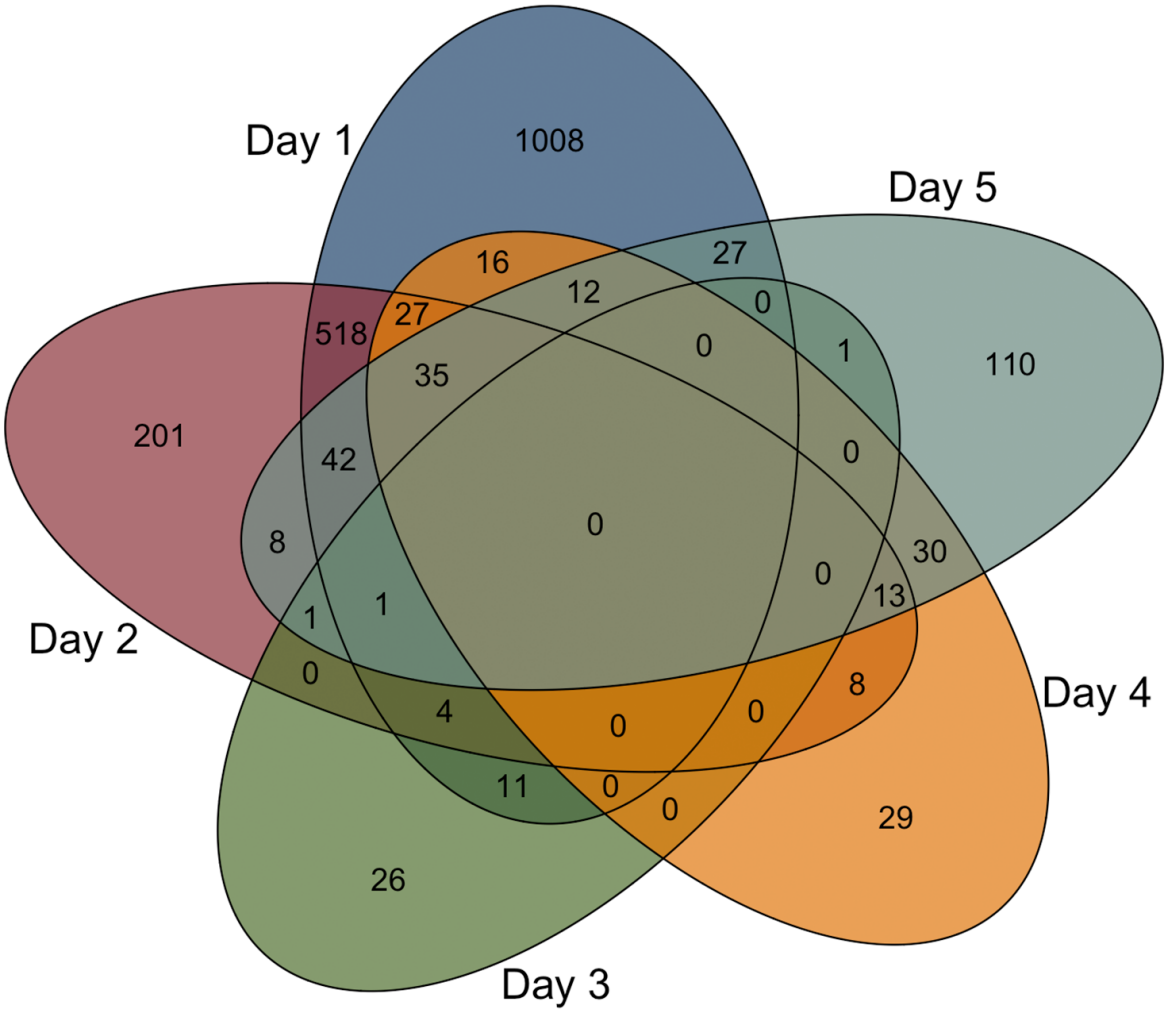
Venn diagram of overlap in significant gene regulation for each day. A Venn diagram of the differentially expressed genes at days 1, 2, 3, 4, and 5 was created to identify the overlap in gene expression.

Venn diagram and fold change-fold change plots were used to identify genes commonly regulated at different time points (Fig 4 and S1 Fig). Fold change-fold change plots indicated gene expression changes at day 1 and 2 were strongly correlated (S1 Fig) with 565 and 52 commonly up-regulated and down-regulated genes, respectively. Gene expression changes at day 4 and 5 showed a similar correlation pattern with 89 commonly up-regulated genes on these two days. Without strong correlation between day 1 or 2 and 4 and 5 we do see some overlap in gene expression with commonly up- and down-regulated genes between these time points (S1 Fig). Enrichment of the overlaps in the venn diagram and fold change-fold change plots indicated that the DEGs that were commonly up-regulated between days 1 and 2, 1 and 4, 1 and 5, 2 and 4, and 2 and 5 were primarily associated with immune system processes (S3 and S4 Tables). Finally, when comparing the DEGs that were down-regulated at day 1 and then up-regulated at days 4 or 5, we see enrichment in cell cycle related genes. This last finding is in agreement with our published work showing increased cell death (apoptosis and autophagy) 1 day after injury and increased cell proliferation (as evidenced by Ki67 staining) on days 4 and 5 [19].

The above analyses identified the 2 major processes of inflammation and repair that follow experimentally induced injury. Inflammatory processes are focused around days 1 and 2, with some processes continuing during the reparative phase, days 3–5. Taken together these data indicated that there were several specific patterns of gene expression associated with lacrimal gland inflammation and repair following injury.

### Epithelial mesenchymal transition in lacrimal gland regeneration

We previously reported that activation of EMT occurs during lacrimal gland repair and regeneration [6]. Thus, we investigated the expression of major EMT regulators such as transcription factors and chromatin remodelers. *Snai1* expression was up-regulated 1.84 fold 1 day post-injury, and *Zeb2* was up-regulated 2.40 fold 2 days post-injury. Expression of the natural antisense transcript of *Zeb2*, known as *Zeb2os*, was up-regulated 2.47 fold 1 day post-injury. *Vimentin* expression was up-regulated 2.69, 3.07, and 2.31 fold 1, 2 and 5, days post injury; respectively. Expression of the chromatin remodeler *Hmga1*, which has been associated with EMT, was up-regulated 2.62 and 1.86 fold 1 and 2 days post-injury, respectively. Another major regulator of EMT is TGF-β1 (*Tgfb1*) [22], whose expression was up-regulated 2.53 and 2.18 fold 1 and 2 days post-injury. TGF-β1 induced EMT is reliant on the expression of *Has2*, an enzyme involved in hyaluronan synthesis [23]. *Has2* gene expression was up-regulated 2.18 fold 2 days post-injury. *Has2* expression was also up-regulated 5 days post-injury.

These data confirmed the involvement of EMT in lacrimal gland repair following experimentally induced injury.

### Novel gene expression in the regenerating lacrimal gland

In order to identify potential genes involved in lacrimal gland regeneration, we investigated genes that were not expressed in the native lacrimal gland (day 0) and then had expression turned on after injury. There were 63 significantly regulated genes identified (see methods), most of which (53) were up-regulated at days 1 and/or 2. Gene set enrichment identified immune related processes with “response to bacterium” (p = 1.44x10^-4^), “cell chemotaxis” (p = 2.48x10^-4^), and “cytokine secretion” (p = 4.64x10^-4^) as the top hits. The remaining genes were not up-regulated until at least day 2 but mostly at day 5, which may implicate them in lacrimal gland regeneration. This included 2 genes involved in re-innervation which were up-regulated 3.25 and 2.23 fold, respectively at day 5: *Tnn*, which is involved in neurite repulsion [24] and *Unc5c*, which is involved in axonal guidance [25, 26]. There are also several genes associated with immune cells associated with B-cell development, plasmacytoid dendritic cell development, and macrophage inflammatory response [27–29]. One of these genes Cd5*l*, encodes a secreted regulator of lipid synthesis that is primarily secreted by macrophages in inflamed tissues and might be involved in resolution of inflammation [30].

Taken together these data identified the expression of novel genes that might be involved in resolution of inflammation and regeneration of the lacrimal gland after experimentally induced injury.

### Identification of molecular signatures

**Fig 5:**
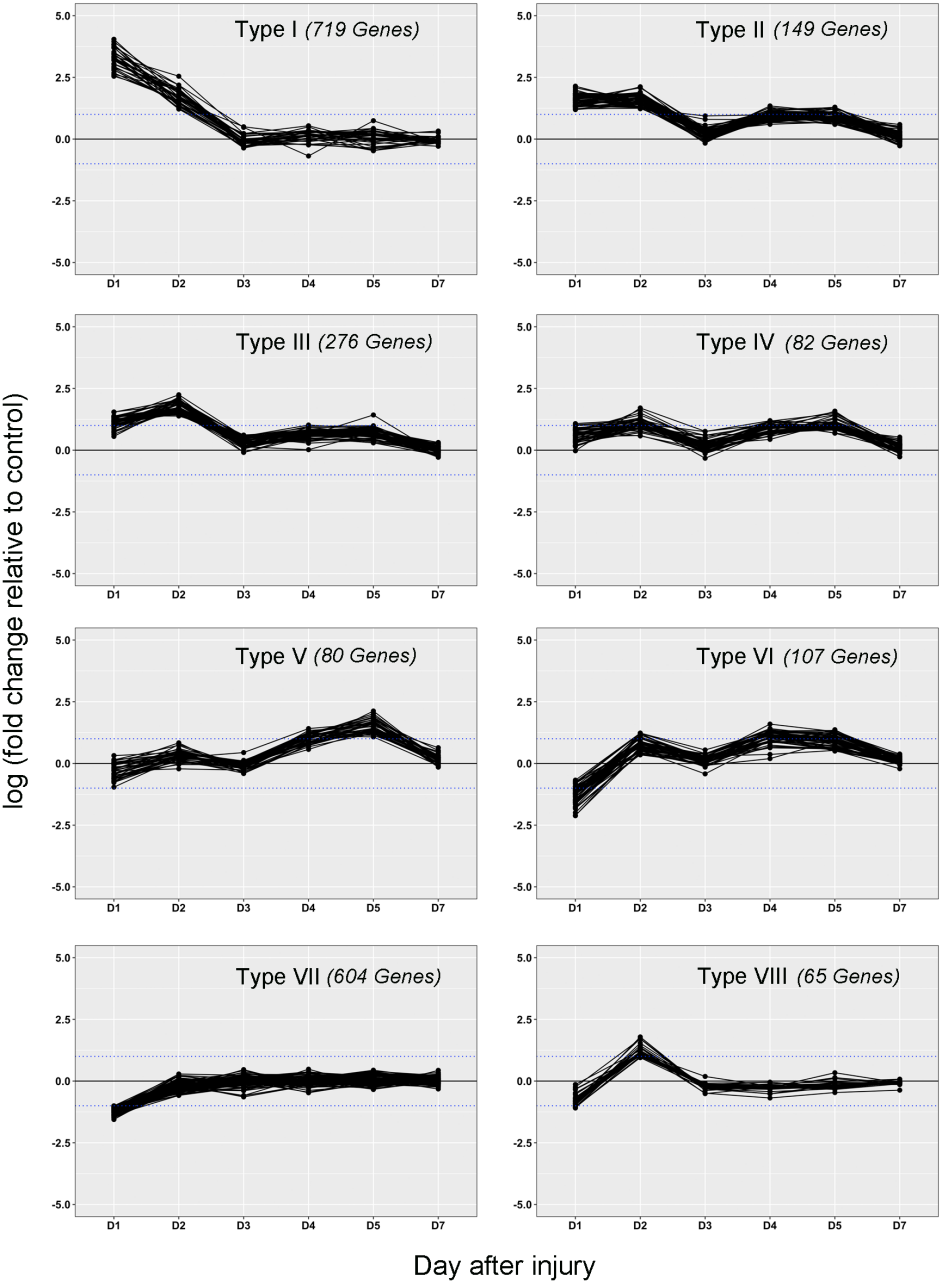
Representative molecular signatures of lacrimal gland inflammation and repair. Expression (log2[fold change]) pattern of clusters representative of the 8 consolidated clusters were plotted for days 1, 2, 3, 4, 5, and 7. Threshold for significant up/down-regulation (+/- 1 = log2[+/-2]) is indicated by dotted blue line.

Clustering of all DEGs according to their expression at each time point was used to identify molecular signatures associated with lacrimal gland inflammatory and repair processes (S2 Fig). Among the resulting 50 clusters, 13 main patterns of expression were identified and defined distinct molecular signatures; this included 8 larger representative clusters (type I-VIII) (displayed in Fig 5) and 5 smaller clusters with unique molecular signatures (type IX-XIII) (S2 Fig). Each of the unique molecular signatures contained 17 or less DEGs while the larger representative clusters contained 65 or more DEGs, with the largest cluster containing 719 DEGs (Table 2, see S5 Table for full cluster expression data). Each of these clusters was further investigated by enrichment of the associated DEGs.

**Table 2:** Summary of identified molecular signatures.

	# of Clusters	# of Genes	Top Hit (BP: GO Term)	p-value
**Type I**	14	719	Immune System Process	1.27E-52
**Type II**	3	149	Immune Response	2.76E-54
**Type III**	6	276	Immune System Process	7.48E-12
**Type IV**	3	82	Cell Cycle	2.93E-05
**Type V**	2	80	Skeletal System Development	2.42E-10
**Type VI**	3	107	Mitotic Cell Cycle Process	5.23E-23
**Type VII**	12	604	Cellular Amino Acid Metabolic Process	7.22E-13
**Type VIII**	2	65	Skeletal Muscle Tissue Development	3.78E-05
**Type IX**	1	11		
**Type X**	1	2		
**Type XI**	1	6		
**Type XII**	1	17		
**Type XIII**	1	13	Complement Activation, Classical Pathway	9.76E-04

DEGs included in clusters I to III were characterized by their up-regulation at early time points following injury (Fig 5). Cluster I genes showed strong up-regulation at day 1 and 2 followed by return to basal gene expression levels at day 3 post injury. Genes from clusters II and III showed a weaker up-regulation at day 1 compared to genes in cluster I. These genes were also weakly upregulated during the reparative phase as well (days 3–5). Gene set enrichment analyses of these clusters indicated that they were associated with the immune response (S6 Table), with enriched categories such as cell chemotaxis, cytokine production, cell activation and regulation of the immune response. This analysis indicated the involvement of the immune response during the early phase post injury (days 1–2) but also during the reparative phase (days 3–5).

**Fig 6:**
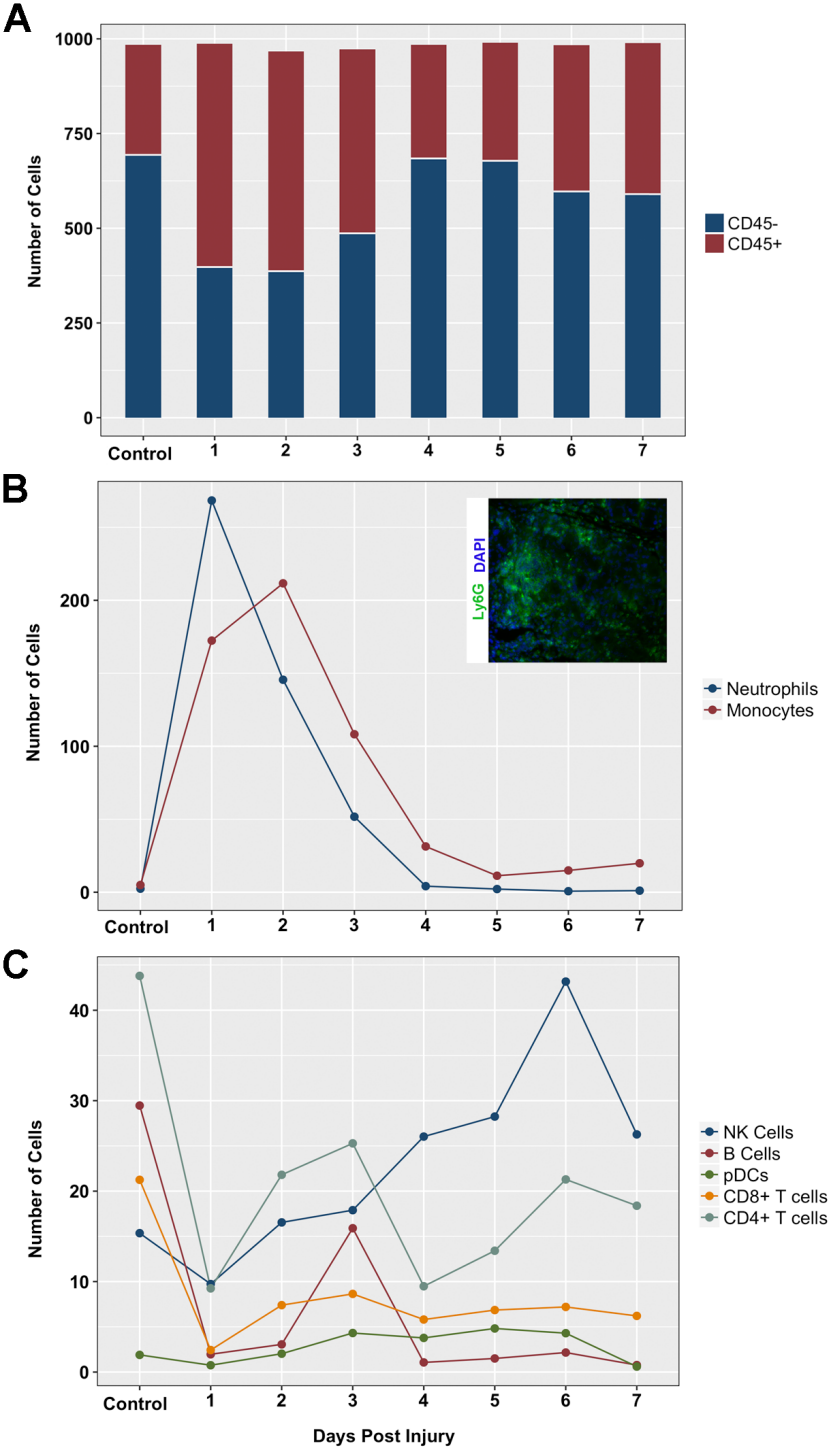
Quantification of immune cell populations. The number of cells per thousand of analyzed cells were plotted by day for (A) CD45+ (immune cells) CD45- (non-immune cells), (B) cells with large population change, monocytes and neutrophils, and (C) cells with small population change, natural killer (NK), B, plasmacytoid dendritic (pDC), CD4+ and CD8+ T cells. Insert shows representative immunofluorescence staining of Ly-6G (neutrophils) 1 day post IL-1α injection.

To further investigate immune subsets involved in the lacrimal gland response to acute injury we performed CyTOF immuno-phenotyping of lacrimal gland single cell suspensions at different time points following injury (for gating see S3 Fig). The absolute number of CD45^+^ hematopoietic cells increased in the lacrimal gland 1 day post-injury and returned to normal levels by day 4 post-injury (Fig 6A). Neutrophil and monocyte populations were the main infiltrating cell subsets at day 1 and 2 post-injury (Fig 6B). Neutrophil infiltration was confirmed by immunofluorescence staining of lacrimal gland sections with an anti-Ly-6G antibody, a marker of neutrophils (see insert Fig 6B for representative staining). The other major immune cell populations that were investigated all showed decreased cell numbers at day 1 after injury followed by slow recovery of cell numbers during the tissue repair period (Fig 6C). Natural killer cell numbers increased over the course of the injury and repair process and peaked between day 4 and 6. Taken together, these data showed that the major infiltrating cells are neutrophils and monocytes, in agreement with the DEGs identified in type I II, and III clusters (S6 Table).

The DEGs associated with clusters IV, V, and VI were characterized by a selective up-regulation during the repair phase (after day 2) (Fig 4). Clusters IV and VI were both enriched in cell cycle-related genes having top category enrichments of “cell cycle” (p = 2.93x10^-5^) and “mitotic cell cycle process” (p = 5.23x10^−23^); respectively (S6 Table). Cluster V contained genes enriched for cellular growth and extracellular matrix organization with a top enriched category of “skeletal system development” (p = 2.4x10^-10^). Taken together we see that up-regulation of genes associated with lacrimal gland regeneration begins immediately following the initial inflammatory response 2 days post injury.

The inflammatory and regenerative processes are clearly major contributors to the DEGs observed in response to acute lacrimal gland injury. However, there are many DEGs that did not fall into either of these categories and were primarily found in the smaller clusters (IX-XIII) and cluster VII. The DEGs that fell into cluster type VII were down-regulated immediately after injury (day 1) and showed slow return to basal levels over the ensuing days (days 2–5) (Fig 5). This cluster contained a large number of DEGs (604, Table 2), which were enriched for processes associated with normal cellular function and response to cellular death; “cellular amino acid metabolic process” (p = 7.22x10^-13^), “endoplasmic reticulum unfolded protein response” (p = 1.28x10^-8^), and “transport” (p = 1.74x10^-4^) (S4 Table). Of the remaining small clusters only one was enriched for any processes, cluster type XIII, which was enriched for “complement activation, classical pathway” (p = 9.76x10^-4^) (S6 Table). This cluster was composed of DEGs that were up-regulated throughout the whole process of inflammation and regeneration (days 1–5; Fig 5). The DEGs in the other clusters (type IX-XII) were predicted genes that contained no functional annotations or were pheromone related genes with no cellular function.

Of the 8 major molecular signatures identified using clustering 6 were clearly attributed to the major processes of inflammation (I-III) and tissue repair (IV-VI). CyTOF immuno-phenotyping was used to identify neutrophils, monocytes, and natural killer cells as major players in response to experimentally induced lacrimal gland injury. To identify the major genes involved in lacrimal gland repair more in depth analysis was required (see below).

### Role of extracellular matrix remodeling in lacrimal gland repair

Re-organization of the extracellular matrix is a major part of the healing and regeneration processes. We therefore examined the expression of specific extracellular matrix associated factors. We investigated the expression of collagen chains (Table 3), matrix modifying enzymes, such as matrix metalloproteinases (MMPs) (Table 4), and other factors associated with the extracellular matrix and its maintenance (Table 5). The up-regulation of collagen chains and enzymes involved in collagen synthesis encoded by *Loxl* and *Sulf* genes occurred almost exclusively at days 4 and 5 with some genes also being down regulated at day 1 (Table 3). Molecules involved in modifying the extracellular matrix showed different patterns of expression with some being up-regulated at days 1 and 2 such as MMP8 (a neutrophil collagenase), MMP9 (another collagenase secreted by immune as well as epithelial cells), MMP3 (stromelysin which besides collagens also degrades fibronectin and laminin), while others were up-regulated at days 4 and 5, the repair phase, such as MMP2, a collagenase; MMP14, a membrane-bound MMP which activates MMP2; and MMP19 which is involved in angiogenesis and lymphocyte extravasation (Table 4). The expression of a number of ADAMs (a disintegrin and metalloproteinase) and ADAMTS (a disintegrin and metalloproteinase with a thrombospondin type 1 motif) was also down-regulated during the acute injury phase (day 1) and up-regulated during the repair phase (days 4 and 5) (Table 4). Of interest, the expression of genes encoding a number of extracellular glycoproteins necessary for ECM remodeling was selectively up-regulated at days 4 and 5 (Table 5). Tenascins, (TN) are a family of ECM glycoproteins which includes five known members TN-C, TN-R, TN-X, TN-Y and TN-W [31, 32]. In contrast to other ECM components, such as fibronectin, TN proteins have anti-adhesive effects [31, 32]. TN-C has been implicated in guidance of migrating neurons and axons, synaptic plasticity and neuronal regeneration; TN-X has been implicated in matrix maturation during wound healing; and TN-N has been shown to mediate neurite repulsion [24, 32]. Our data showed the selective upregulation of TN-C and TN-N at day 5, and TN-X at days 4 and 5-post injury suggesting the involvement of these glycoproteins in re-innervation of the newly formed acinar cells. Indeed, the lacrimal gland is heavily innervated by parasympathetic nerves as well as sympathetic and sensory ones (albeit less compared to the parasympathetic innervation) [3]. These nerves play important roles in tissue development but also in controlling lacrimal gland secretion [3, 4].

**Table 3:** Differential expression of collagen chains by day.

	Time Post Injury (days)							
Gene Name	1	2	3	4	5	7	14	14S
*Col1a1*	-1.7	1.2	-1.1	**2.2**	**4.0**	1.1	-1.0	-1.2
*Col1a2*	-1.6	1.2	-1.1	**2.2**	**3.7**	1.2	-1.1	-1.2
*Col3a1*	-1.3	1.3	-1.1	**2.4**	**3.4**	1.2	-1.1	-1.2
*Col5a1*	-1.7	1.1	-1.0	**2.3**	**3.8**	1.2	-1.1	-1.3
*Col5a2*	-1.6	1.3	-1.1	**2.3**	**3.6**	1.2	-1.1	-1.2
*Col5a3*	-1.6	1.0	-1.3	1.9	**2.1**	1.1	1.1	-1.1
*Col6a1*	-1.7	-1.1	-1.1	1.9	**2.4**	1.1	-1.0	-1.2
*Col6a2*	-1.6	-1.0	1.0	1.8	**2.3**	1.0	-1.1	-1.1
*Col6a3*	-1.3	1.2	-1.1	1.8	**2.3**	1.0	-1.0	-1.1
*Col6a5*	**-2.8**	1.0	1.2	**2.0**	**2.7**	1.1	-1.1	-1.2
*Col6a6*	**-2.5**	1.0	-1.0	1.9	1.8	1.0	-1.0	-1.0
*Col8a1*	1.2	1.5	-1.2	-1.1	**2.4**	1.0	1.0	-1.1
*Col11a1*	-2.0	-1.1	-1.1	-1.1	**3.6**	-1.0	-1.0	-1.1
*Col12a1*	-1.2	1.5	-1.2	1.0	**4.0**	-1.1	-1.2	-1.1
*Col14a1*	**-2.9**	-1.0	-1.1	1.9	**2.3**	1.1	-1.1	-1.0
*Col16a1*	-1.5	1.1	1.0	1.8	**2.4**	1.4	1.0	1.1
*Col17a1*	1.4	1.9	1.2	1.8	**2.0**	1.2	1.1	1.1
*Col18a1*	1.6	1.8	1.2	1.5	**2.4**	1.1	1.0	-1.2
*Loxl1*	1.2	1.5	-1.0	1.9	**2.1**	1.0	-1.1	-1.3
*Loxl2*	1.1	1.9	1.1	**2.3**	**2.8**	1.2	-1.1	-1.2
*Sulf1*	-1.0	1.4	-1.0	1.7	**2.6**	1.1	1.0	-1.4
*Sulf2*	1.5	**2.1**	1.1	1.6	**2.1**	1.2	1.0	1.2

Bold text indicates significant up-/down-regulation.

**Table 4:** Differential expression of matrix modifying enzymes by day.

	Time Post Injury (days)							
Gene Name	1	2	3	4	5	7	14	14S
*Mmp2*	-1.5	1.1	-1.1	1.9	**2.5**	1.2	1.0	-1.1
*Mmp3*	**3.0**	**2.1**	-1.7	1.4	1.9	1.0	-1.1	-1.0
*Mmp8*	**13.1**	**2.5**	-1.1	1.0	1.1	1.0	-1.1	-1.1
*Mmp9*	**5.9**	**4.1**	1.2	1.5	1.5	-1.1	-1.4	-1.3
*Mmp14*	1.1	1.5	-1.1	**2.1**	**3.0**	1.1	1.2	-1.1
*Mmp19*	**3.8**	**2.6**	-1.1	**2.2**	**2.4**	-1.0	-1.4	-1.4
*Mmp23*	**-2.4**	1.1	1.1	1.9	**2.2**	1.0	-1.1	-1.2
*Mmp25*	**2.1**	1.4	-1.2	-1.1	-1.0	1.1	1.0	-1.1
*Timp1*	**4.0**	**2.7**	-1.2	1.2	**2.3**	-1.0	-1.2	-1.2
*Adam8*	**8.7**	**3.7**	-1.1	2.0	1.8	-1.1	-1.3	-1.3
*Adam11*	**-2.7**	-1.2	-1.1	1.1	1.1	1.0	1.1	-1.1
*Adam12*	-1.2	1.2	-1.0	**2.3**	**3.7**	-1.1	-1.1	-1.2
*Adam15*	**2.0**	1.7	1.1	1.4	1.5	1.0	1.1	1.1
*Adam19*	1.1	1.5	1.0	1.9	**2.3**	1.0	1.1	-1.0
*Adam22*	**-2.1**	1.0	1.0	-1.1	1.2	1.0	-1.0	-1.1
*Adamts1*	**2.7**	2.0	1.0	1.5	1.8	1.1	-1.3	-1.1
*Adamts2*	-2.0	-1.1	-1.2	**2.0**	**2.7**	1.2	1.0	-1.1
*Adamts4*	**3.4**	1.6	-1.2	1.4	**2.7**	-1.1	-1.1	-1.2
*Adamts7*	1.3	1.9	-1.1	1.3	**2.2**	-1.2	-1.1	-1.1
*Adamts12*	**-2.7**	1.0	-1.2	**2.1**	**2.5**	1.3	-1.0	-1.0
*Adamts14*	-1.2	1.5	1.1	**2.2**	1.4	1.1	-1.2	-1.2
*Adamts16*	**-2.7**	-1.5	1.1	1.1	1.5	1.0	-1.0	1.0
*Adamts17*	**-2.6**	-1.1	-1.2	1.1	1.3	-1.0	1.1	1.1
*Adamtsl2*	**-2.8**	-1.2	1.1	1.5	1.3	-1.1	-1.0	-1.0

Bold text indicates significant up-/down-regulation.

**Table 5:** Differential expression of factors associated with the extracellular matrix and its maintenance by day.

	Time Post Injury (days)							
Gene Name	1	2	3	4	5	7	14	14S
*Fbln1*	-2.0	-1.0	-1.3	1.4	**2.2**	1.1	1.1	-1.0
*Fbln2*	-1.5	1.2	-1.0	1.8	**3.0**	1.0	1.0	1.0
*Fbln7*	**-3.6**	-1.5	1.0	1.2	1.6	1.1	1.3	1.2
*Thbs1*	**4.0**	**3.6**	-1.0	1.2	**2.1**	1.0	-1.2	-1.4
*Thbs2*	**-2.3**	-1.1	1.0	1.9	**3.8**	1.1	-1.1	-1.1
*Thbs3*	-1.2	-1.2	-1.2	**2.1**	**2.4**	1.1	-1.0	-1.3
*Lama1*	**-2.4**	-1.1	1.2	1.2	1.2	-1.0	-1.0	-1.1
*Lama2*	**-3.3**	-1.6	-1.2	1.2	1.2	-1.0	1.0	1.0
*Lama4*	1.2	1.5	1.1	2.0	**2.2**	-1.0	-1.0	-1.2
*Tnxb*	-1.6	-1.0	1.1	**2.2**	**2.1**	-1.1	-1.1	-1.1
*Tnn*	1.0	-1.2	1.0	-1.0	**3.2**	1.0	-1.0	1.0
*Tnc*	1.1	1.8	-1.2	1.0	**2.9**	-1.1	1.0	-1.2
*Mfap2*	-2.0	1.1	-1.2	1.6	**2.5**	1.1	-1.1	-1.2
*Mfap4*	**-5.2**	**-2.3**	-1.3	**2.5**	**4.7**	1.4	1.3	-1.2
*Mfap5*	**-2.2**	-1.3	-1.3	1.9	**2.3**	1.4	-1.3	-1.4
*Sdc3*	**4.4**	**4.4**	1.0	**2.2**	1.9	-1.0	-1.2	-1.2
*Tgfb1*	**2.5**	**3.2**	1.1	1.6	1.4	1.0	-1.1	-1.1
*Ltbp1*	-1.3	-1.0	-1.2	1.4	**2.0**	1.1	1.1	-1.0
*Ltbp2*	1.1	1.3	1.4	1.1	**2.5**	-1.2	-1.1	-1.1
*Postn*	-1.4	-1.0	-1.0	1.8	**3.0**	1.2	-1.0	-1.2
*Fscn1*	**2.6**	**2.1**	-1.2	1.4	**2.8**	1.0	1.1	-1.1
*Sh3pxd2b*	1.8	1.7	1.1	1.9	**2.2**	1.2	-1.0	-1.2
*Serpinh1*	1.7	1.5	1.0	2.0	**2.7**	1.1	-1.2	-1.4
*Bgn*	-1.2	1.4	1.0	2.0	**3.3**	1.0	1.1	-1.0
*Eln*	-1.9	-1.1	-1.2	1.6	**2.7**	1.1	1.1	-1.2
*Lum*	-1.7	1.1	-1.0	1.5	**2.0**	1.2	-1.1	-1.0
*Fn1*	1.3	1.3	-1.1	**2.2**	**2.4**	-1.1	-1.1	-1.3

Bold text indicates significant up-/down-regulation.

The embryonic lacrimal gland, similar to the salivary and mammary glands and lungs, develops through a process termed branching morphogenesis [33–35]. This process involves repetitive formation of epithelial clefts and buds that invade the surrounding ECM [35]. Most of the ECM components identified in our studies are crucial for branching morphogenesis providing not only structural stability (collagens, fibronectin, elastin, etc.) to guide unidirectional tissue expansion but also by triggering other processes such neovascularization or re-innervation (tenascins, etc.) which are important for proper lacrimal gland development [33, 35]. Lastly, the ECM serves as a reservoir for growth factors which can be proteolytically cleaved (by activated MMPs) and released locally to stimulate proliferation and branching morphogenesis [36–39].

### Identification of muscle-protein expressing cells

Cluster VIII contained 65 DEGs (Table 2) that were up-regulated at day 2 and showed normal expression immediately before (day 1) and immediately after (day 3; Fig 5). Genes in Cluster VIII were associated with muscle related processes such as “skeletal muscle tissue development” (p = 3.78x10^-5^), “muscle system process” (p = 5.35x10^-4^) and “muscle structure development” (p = 8.9x10^-4^) (S6 Table). Among the 17 enriched genes that were associated with these processes, fold changes at day 2 ranged from 1.9 for *Kelch-like 41* to 3.5 for *Sarcalumenin* (Table 6). Of these 17 genes, 3 were myosin-related, *Mylk2*, *Myo18b*, and *Mybpc2*, and encoded proteins that are commonly found in muscle tissue where they facilitate muscle cell contraction. These 3 genes were up-regulated by 2.9, 2.3, and 2.2-fold 2 days post injury, respectively (Table 6). There were also several other enriched genes that encoded proteins specific to muscle cells such as *des* (desmin), *Ryr1* (ryanodine receptor 1), and *Srl* (sarcalumenin) which were up-regulated 3.3, 2.7, and 3.5-fold 2 days post-injury, respectively (Table 6). The larger representative cluster (VIII) also included *Txlnb* which encodes β-taxilin, another muscle specific gene which was not picked up in the enrichment, and whose expression was up-regulated 3.3-fold 2 days post injury.

**Table 6:** Muscle-related genes from cluster type VIII.

Gene	Full Gene Name	Fold Change	p-value
*Srl*	Sarcalumenin	3.46	3.48E-07
*Des*	Desmin	3.27	1.04E-06
*Mylk2*	Myosin, Light Polypeptide Kinase 2, Skeletal Muscle	2.91	2.43E-06
*Ryr1*	Ryanodine Receptor 1, Skeletal Muscle	2.72	8.09E-06
*Ldb3*	LIM Domain Binding 3	2.54	2.86E-05
*Cacna1s*	Calcium Channel, Voltage-Dependent, L Type, Alpha 1S Subunit	2.54	1.00E-04
*Pgam2*	Phosphoglycerate Mutase 2	2.28	1.70E-03
*Hlx*	H2.0-like Homeobox	2.27	4.00E-04
*Myo18b*	Myosin XVIIIb	2.26	3.10E-05
*Mybpc2*	Myosin Binding Protein C, Fast-Type	2.21	8.87E-05
*Rcsd1*	RCSD Domain Containing 1	2.18	5.83E-06
*Smyd1*	SET and MYND Domain Containing 1	2.13	1.90E-03
*Mef2c*	Myocyte Enhancer Factor 2C	2.11	2.38E-08
*Asb2*	Ankyrin Repeat and SOCS Box-Containing 2	2.08	2.70E-03
*Klhl31*	Kelch-like 31	2.02	3.00E-04
*Smpx*	Small Muscle Protein, X-linked	1.98	6.50E-03
*Klhl41*	Kelch-like 41	1.93	1.38E-02

**Fig 7:**
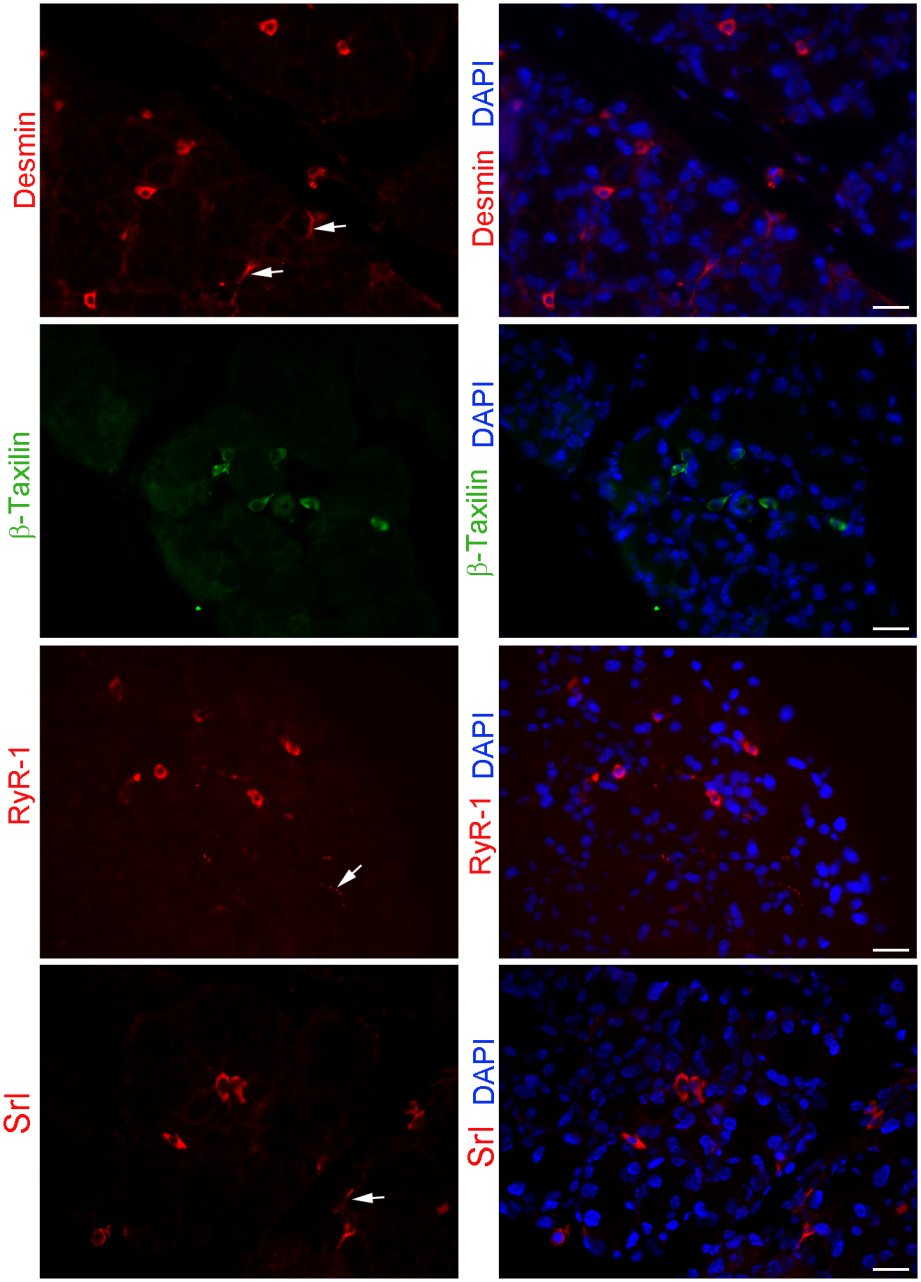
Immunohistochemical analysis of muscle related proteins in 2 day post injury lacrimal glands. Lacrimal gland tissue from IL-1α injected mice were stained for muscle related proteins desmin, β-taxilin, ryanodine receptor 1 (Ryr-1), and sarcalumenin (Srl). Panels represent staining from 2 days post IL-1α injected lacrimal glands which were counterstained with DAPI to visualize cell nuclei. Scale bars represent 25 μm.

**Fig 8:**
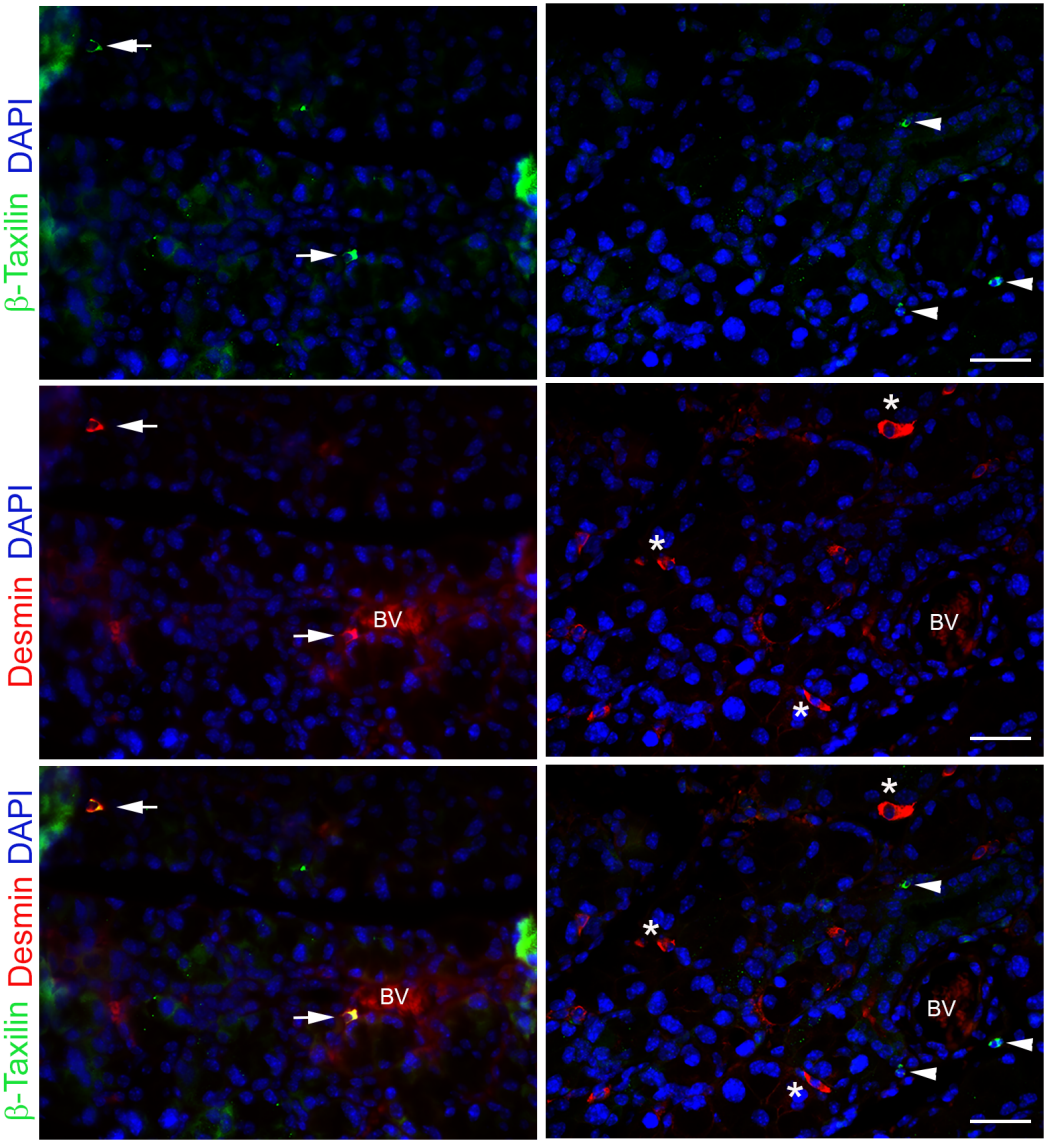
Double-staining of β-taxilin and desmin proteins. Lacrimal gland tissue from IL-1α injected mice were double-stained for muscle related proteins desmin and β-taxilin. Panels represent staining from 2 days post IL-1α injected lacrimal glands which were counterstained with DAPI to visualize cell nuclei. Arrows represent cells expressing both proteins; arrowheads represent cells expressing β-taxilin only; stars represent cells expressing desmin only; BV represents a blood vessel. Scale bars represent 25 μm.

To identify the cells expressing these genes we performed immunostaining using antibodies against desmin, β-taxilin, ryanodine receptor 1, and sarcalumenin using lacrimal glands sections prepared from 2-day injected mice. As shown in Fig 7, all 4 antibodies stained round-shaped cells that were present in the stroma between the acini. In addition, there was occasional staining of cells at the base of the acini that was either continuous (desmin and sarcalumenin) or punctate (ryanodine receptor 1) (Fig 7, arrows). The stained cells looked very similar, suggesting that they likely identified the same cell type. To test this hypothesis we performed double-labeling experiments using an antibody against β-taxilin and one against desmin. As shown in Fig 8, in some sections cells were double-stained with β-taxilin and desmin antibodies (highlighted by arrows), whereas in other sections β-taxilin and desmin staining did not co-localize (highlighted by arrowheads and stars). These data suggest that there is a heterogenous population of cells expressing these muscle-related genes.

**Fig 9:**
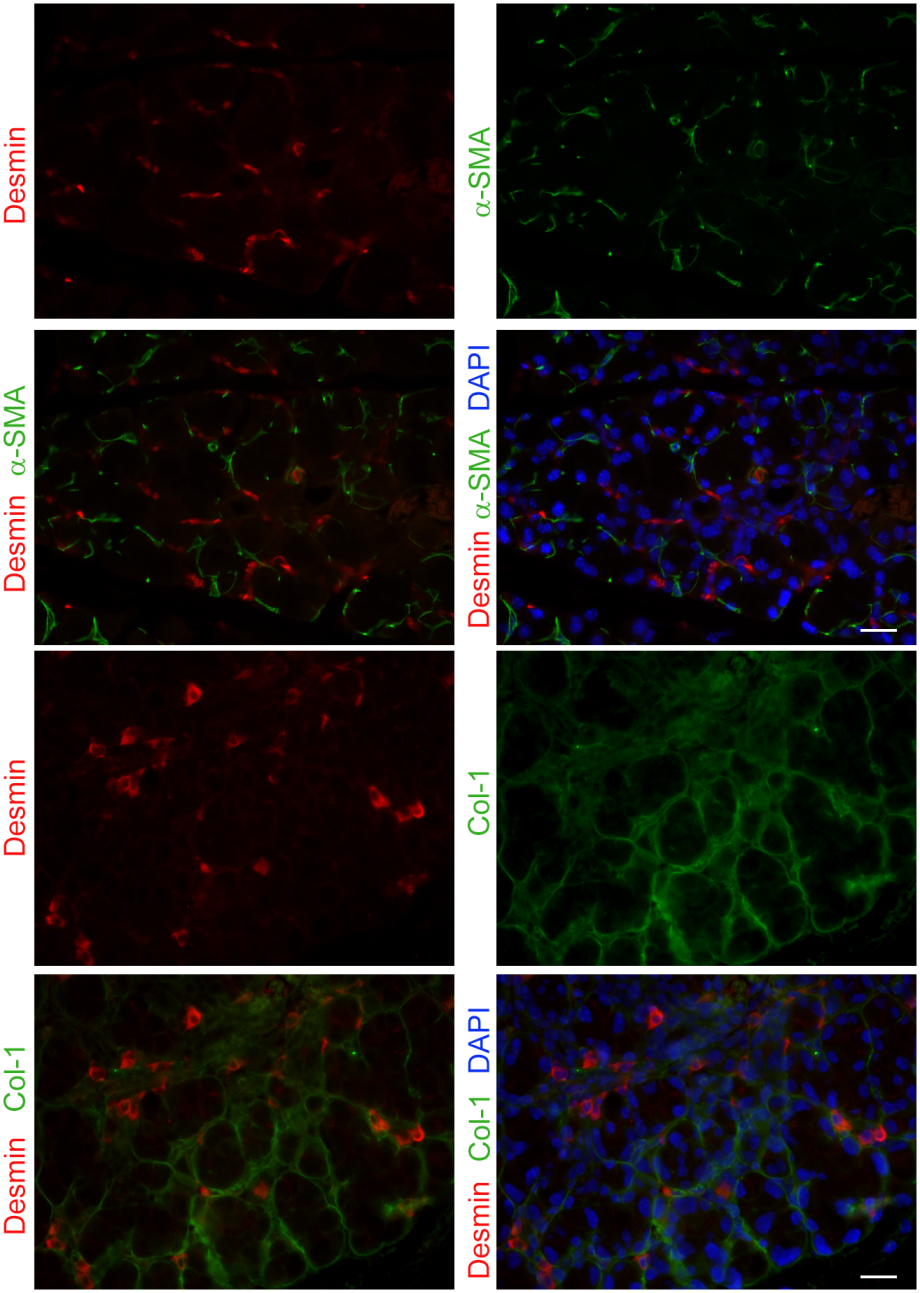
Double-staining of desmin and the myoepithelial cell marker, α-smooth muscle actin. Lacrimal gland tissue from IL-1α and saline (vehicle) injected mice were double stained for desmin and α-smooth muscle actin (α-SMA) or desmin and collagen I (Col-I). Panels represent staining from 2 days post IL-1α injected lacrimal glands which were counterstained with DAPI to visualize cell nuclei. Scale bars represent 25 μm.

In the lacrimal gland the only cells that are known to express muscle proteins are the myoepithelial cells [40]. We therefore performed immunofluorescence staining to visualize the localization of these muscle-specific proteins within the lacrimal gland (Fig 9). Lacrimal gland sections were double stained with antibodies against α-smooth muscle actin (SMA), a marker of myoepithelial cells [40], and desmin. As shown in Fig 9, desmin and SMA staining do not co-localize suggesting that cells other than myoepithelial cells express desmin. We also double-stained lacrimal gland sections for desmin and collagen 1 (which is secreted in part by the myoepithelial cells). As shown in Fig 9, there was minimal co-localization of desmin positive and collagen-1 staining. Similarly, Ryr1 does not seem to be expressed by the myoepithelial cells (data not shown). Please note that the expression of these proteins could be detected, albeit to a lower level, in sections from untreated lacrimal glands as well as those from other time points post-injury (S4 Fig). Future studies are needed to identify/characterize the cells expressing these muscle-related proteins.

## Conclusion

The data from the present studies demonstrated that experimentally induced injury to the lacrimal gland leads to a massive influx of innate immune cells, namely neutrophils followed shortly after by monocytes. In contrast, cells of the adaptive immune system (B and T cells) do not seem to be involved in this process. These findings are reminiscent of the immune response to radiation-induced injury to the lacrimal and salivary glands [41–43]. Several studies showed increased expression of IL-1β following irradiation [44, 45] and established this cytokine as an irradiation-induced stromal growth factor [46]. Our data showed that this cytokine is up-regulated 10- and 6-fold at day 1 and 2 post injury. Our data also show and/or confirm intensive remodeling of the ECM, increased cell proliferation, and activation of EMT during the regenerative phase (4–5 days post injury) of the lacrimal gland. Finally, we identified a group of cells, distinct from the myoepithelial cells, which expressed muscle-related proteins. These cells might play a role in lacrimal gland regeneration, a hypothesis that we plan to investigate in future experiments.

## Supporting information

S1 Table RNA integrity values of samples used for RNA sequencing. RNA integrity values were determined for all samples used in RNA-seq, using a Bioanalyzer 2100, to ensure that the quality of the RNA was sufficient for sequencing.
(XLSX)

S2 Table Gene set enrichment analysis of differentially expressed genes by day. All differentially expressed genes for days 1, 2, 4, and 5, were subjected to gene set enrichment analysis of biological process gene ontology terms. No data were generated for days 3, 7, 14 due to the small number of differentially expressed genes.
(XLSX)

S3 Table Gene set enrichment analysis of commonly differentially expressed genes from Venn diagram. Genes that were found to be commonly differentially expressed using Venn diagrams were subjected to gene set enrichment analysis of biological process gene ontology terms.
(XLSX)

S4 Table Gene set enrichment analysis of commonly differentially expressed genes between time points. Genes that were commonly differentially expressed between 2 time points, as determined using fold change-fold change plots, were subjected to gene set enrichment analysis of biological process gene ontology terms.
(XLSX)

S5 Table Expression data for genes in the 13 identified molecular signatures. The full expression data for all of the genes in each of the 13 identified molecular signatures.
(XLSX)

S6 Table Gene set enrichment analysis of 13 identified molecular signatures. Genes associated with the 13 identified molecular signatures were subjected to gene set enrichment analysis of biological process gene ontology terms.
(XLSX)

S1 Fig Fold change-fold change plots comparing commonly differentially expressed genes between time points. Fold change at 2 time points were plotted against each other to identify commonly differentially expressed genes. The number of genes that fall into each set are shown and the blue arrows indicate the regulation of the genes within a particular set.
(TIF)

S2 Fig Clustering of differentially expressed genes. K-means clustering (k = 50) was used to cluster genes based on expression pattern across all time points. Annotations indicate the type of molecular signature it was assigned to. Threshold for significant up/down-regulation (+/- 1 = log2[+/-2]) indicated by dotted blue line.
(TIF)

S3 Fig Gating of mass cytometry (CyTOF immunophenotyping) data to identify immune cell populations. Mass cytometry data was sequentially gated to identify the specific immune cells populations; percentages of plotted cells not total cells analyzed. Each column represents all cells from a specific gate: column 1 = gating of all single cells, column 2 = gating of CD45^+^ cells, column 3 = gating of non-T non-B cells, and column 4 = gating of CD11b high cells.
(TIF)

S4 Fig Double-staining of desmin and myoepithelial cell marker proteins. Lacrimal glands from 1 day post IL-1α injection and control non-injected mice were double stained for desmin and α-smooth muscle actin (α-SMA), a marker of myoepithelial cells, and counterstained with DAPI to visualize cell nuclei. Scale bar represents 25 μm for all panels.
(TIF)
