# Supplementary figures and images for: RNA-Seq and CyTOF immuno-profiling of regenerating lacrimal glands identifies a novel subset of cells expressing muscle-related proteins
Source: PLoS One. 2017 Jun 29;12(6):e0179385. doi: 10.1371/journal.pone.0179385 (PMC5491009; doi:10.1371/journal.pone.0179385)

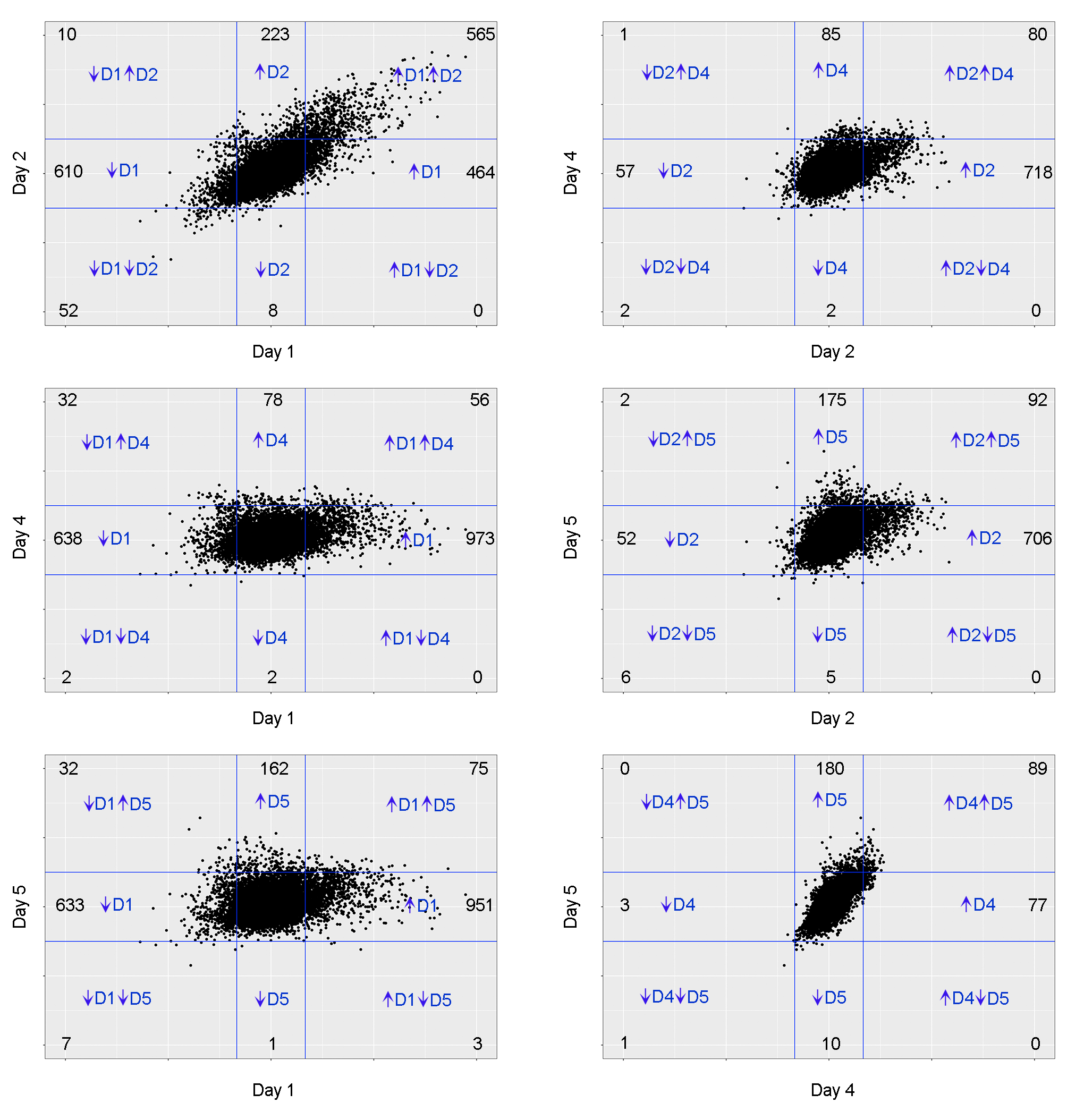

Supplement: S1 Fig — Fold change at 2 time points were plotted against each other to identify commonly differentially expressed genes. The number of genes that fall into each set are shown and the blue arrows indicate the regulation of the genes within a particular set. (TIF) [file pone.0179385.s007.tif]

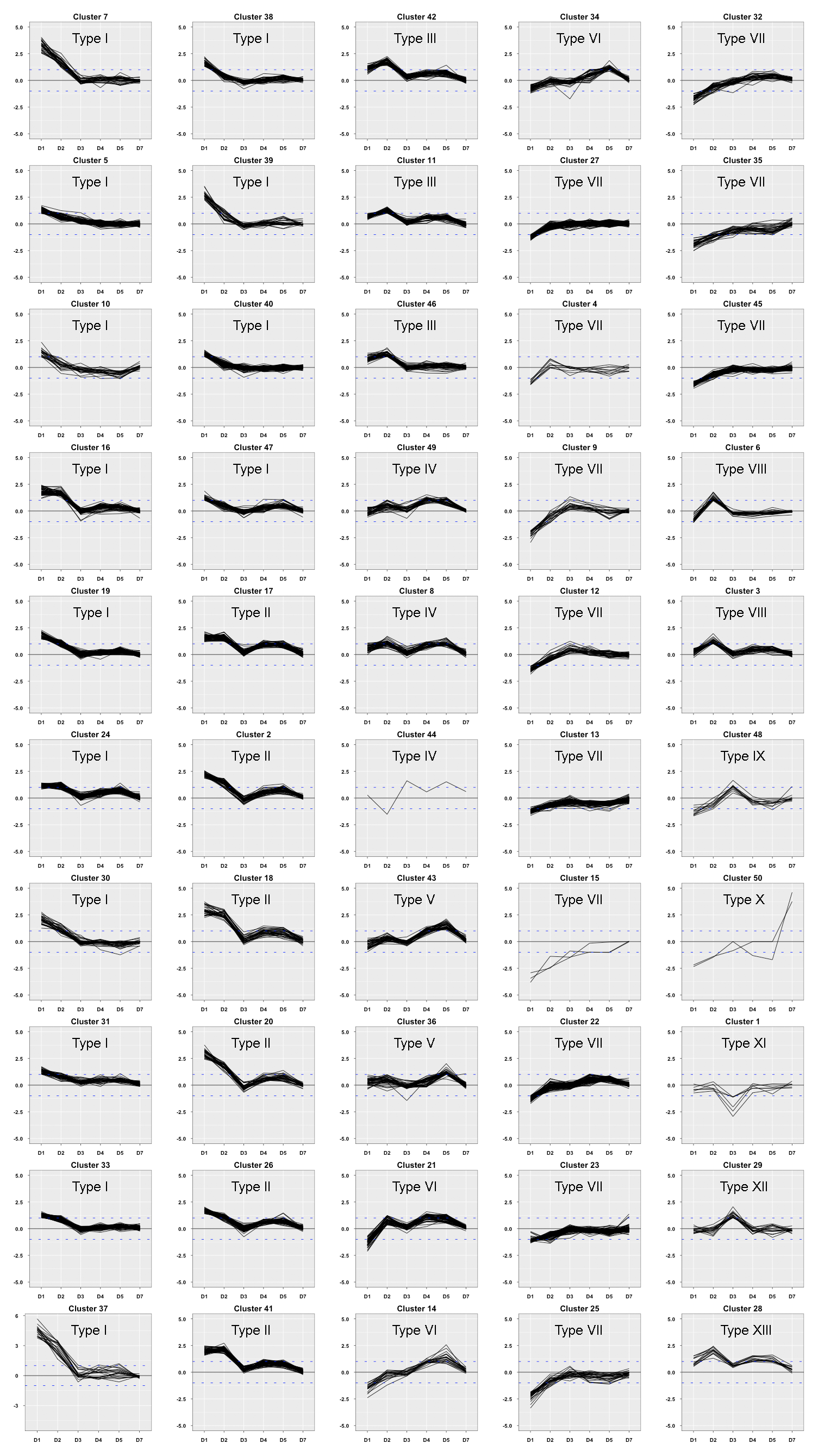

Supplement: S2 Fig — K-means clustering (k = 50) was used to cluster genes based on expression pattern across all time points. Annotations indicate the type of molecular signature it was assigned to. Threshold for significant up/down-regulation (+/- 1 = log2[+/-2]) indicated by dotted blue line. (TIF) [file pone.0179385.s008.tif]

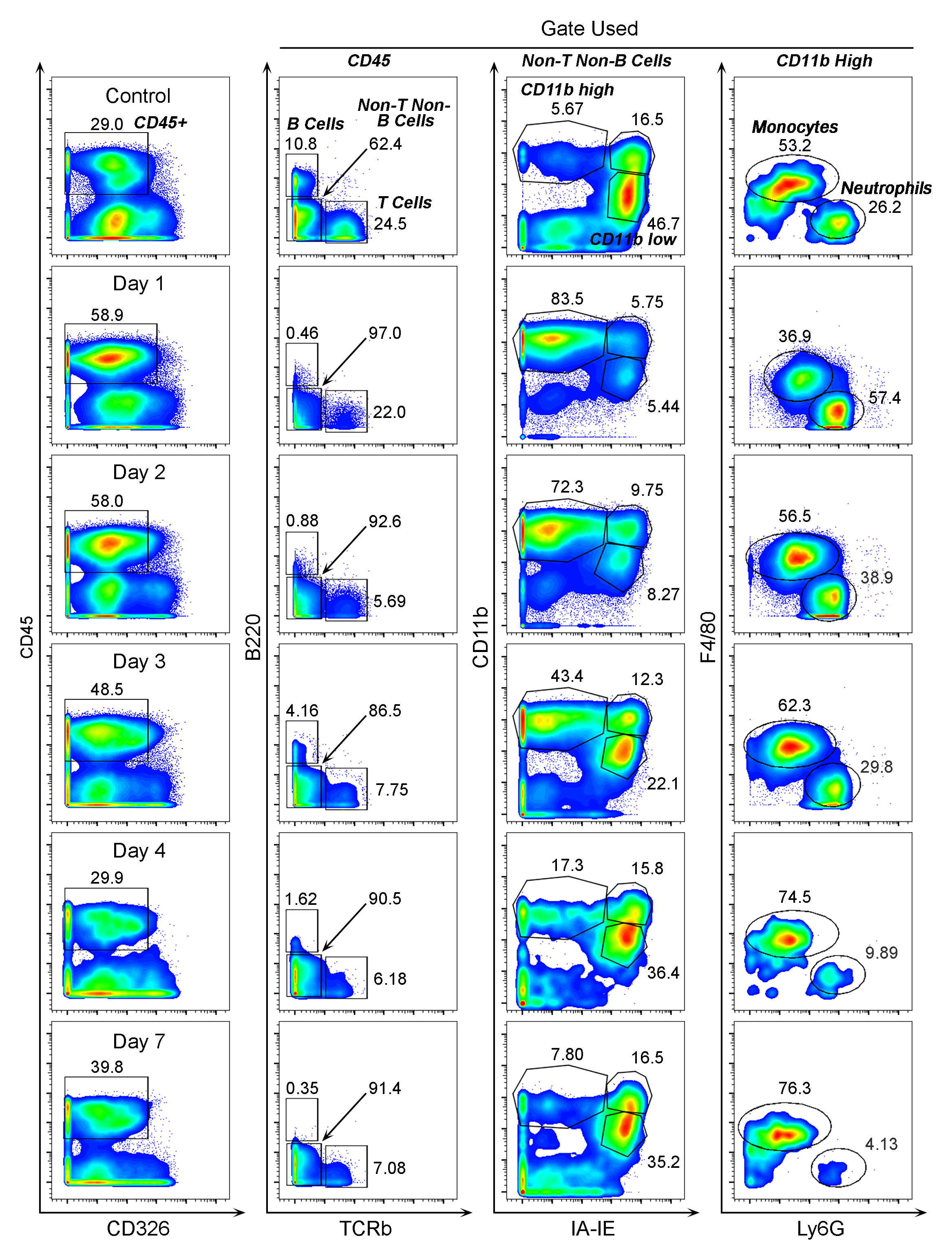

Supplement: S3 Fig — Mass cytometry data was sequentially gated to identify the specific immune cells populations; percentages of plotted cells not total cells analyzed. Each column represents all cells from a specific gate: column 1 = gating of all single cells, column 2 = gating of CD45+ cells, column 3 = gating of non-T non-B cells, and column 4 = gating of CD11b high cells. (TIF) [file pone.0179385.s009.tif]

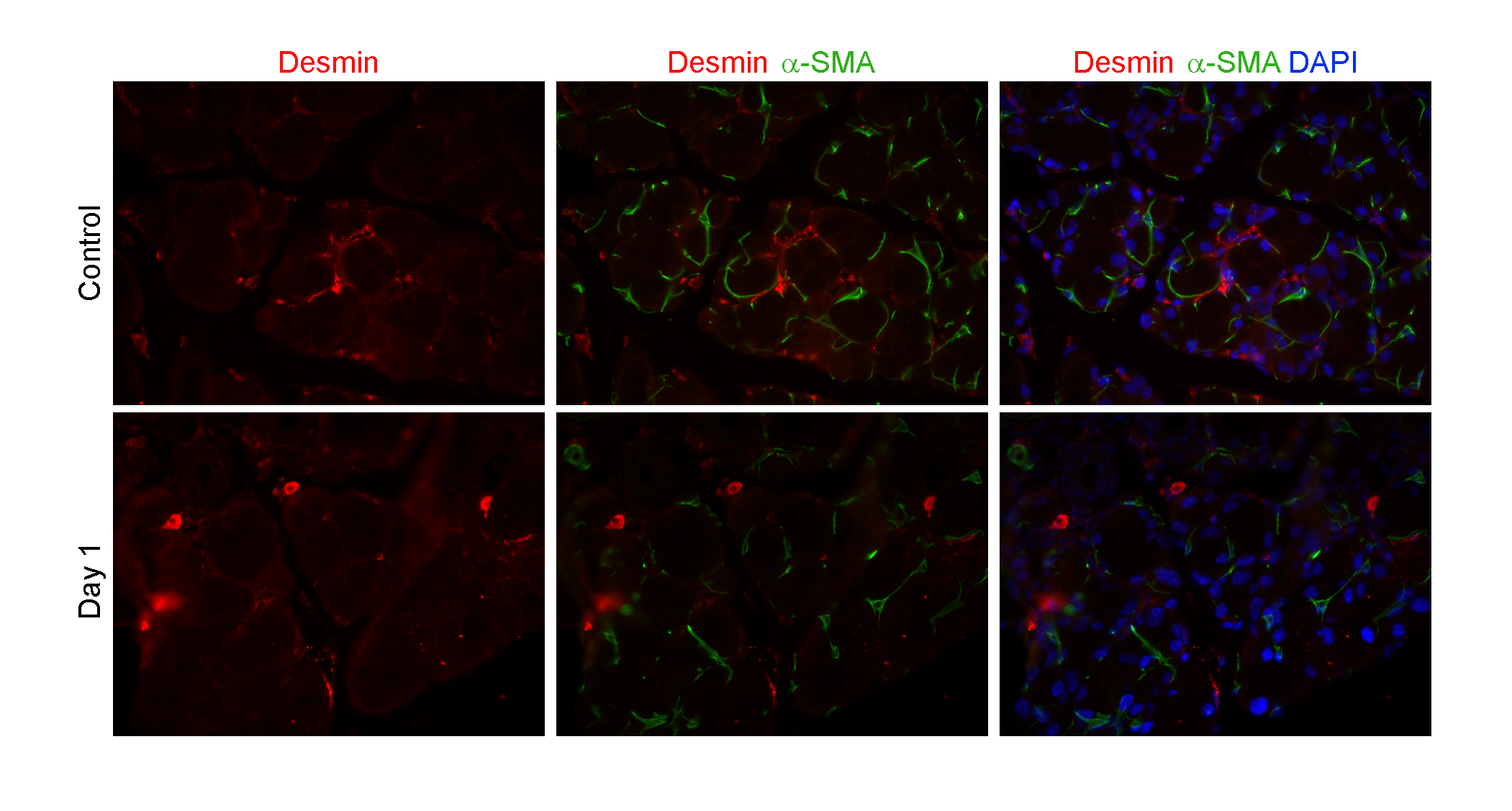

Supplement: S4 Fig — Lacrimal glands from 1 day post IL-1α injection and control non-injected mice were double stained for desmin and α-smooth muscle actin (α-SMA), a marker of myoepithelial cells, and counterstained with DAPI to visualize cell nuclei. Scale bar represents 25 μm for all panels. (TIF) [file pone.0179385.s010.tif]
